# Supplementary material for: A Novel Variant of Deformed Wing Virus (DWV) from the Invasive Honeybee Apis florea (Apidae, Hymenoptera) and Its Ectoparasite Euvarroa sinhai (Acarina, Mesostigmata) in Taiwan
Source: Insects. 2023 Jan 18;14(2):103. doi: 10.3390/insects14020103 (PMC9958760; doi:10.3390/insects14020103)
Supplement: Supplementary file 1 [file insects-14-00103-s001.zip › insects-2107901-SI.pdf]

Supplementary Materials:

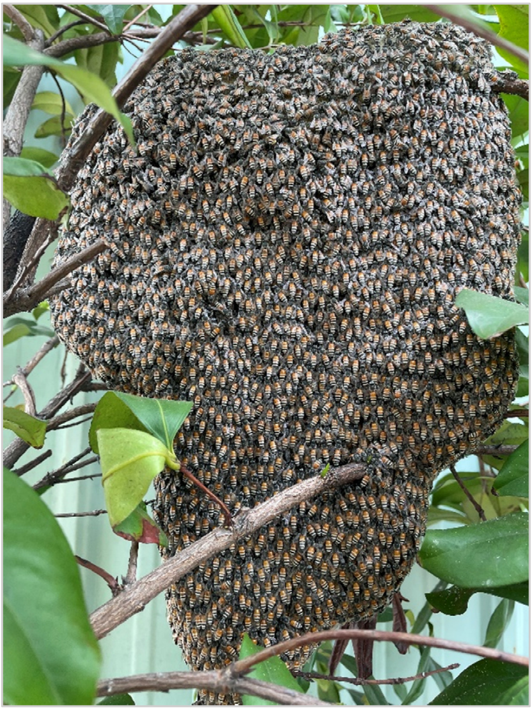

Figure S1. An open-nest of *Apis florea*.

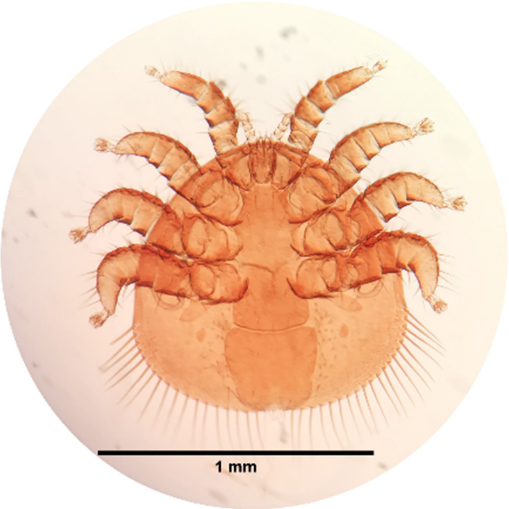

Figure S2. Ventral view of an *Euvarroa sinhai* (scale = 1 mm). (photo by C.-H., Chao)

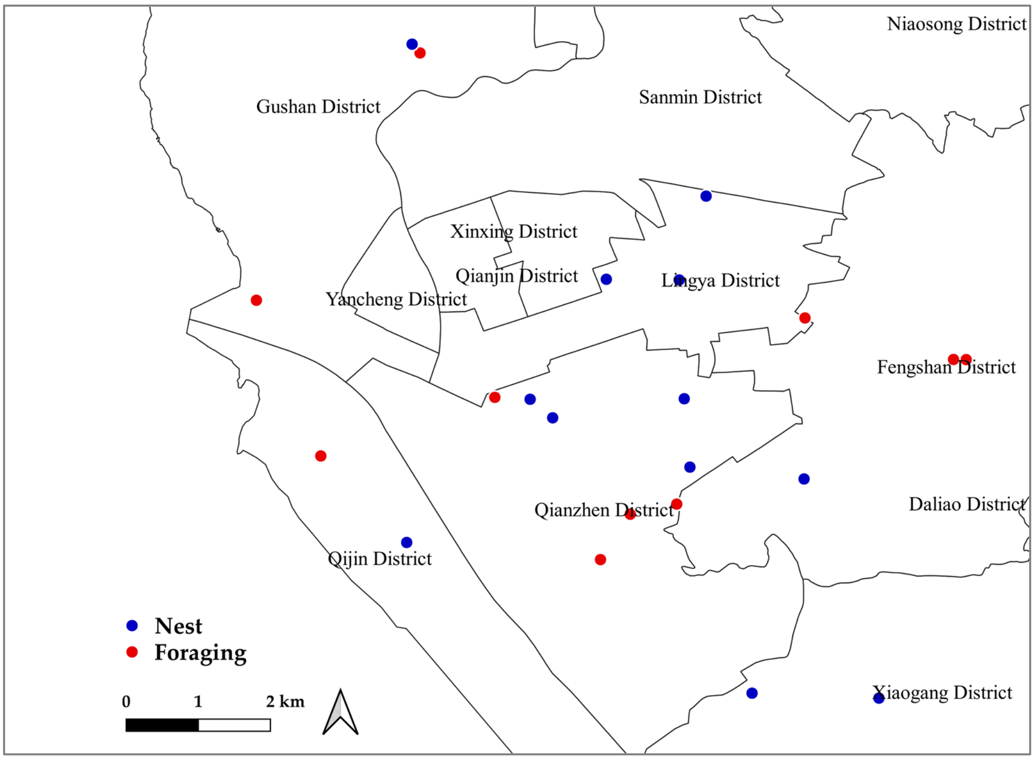

Figure S3: Sampling locations of *A. florea* in Kaohsiung City. Sample collected from nest and open space are shown in blue and red spot, respectively.

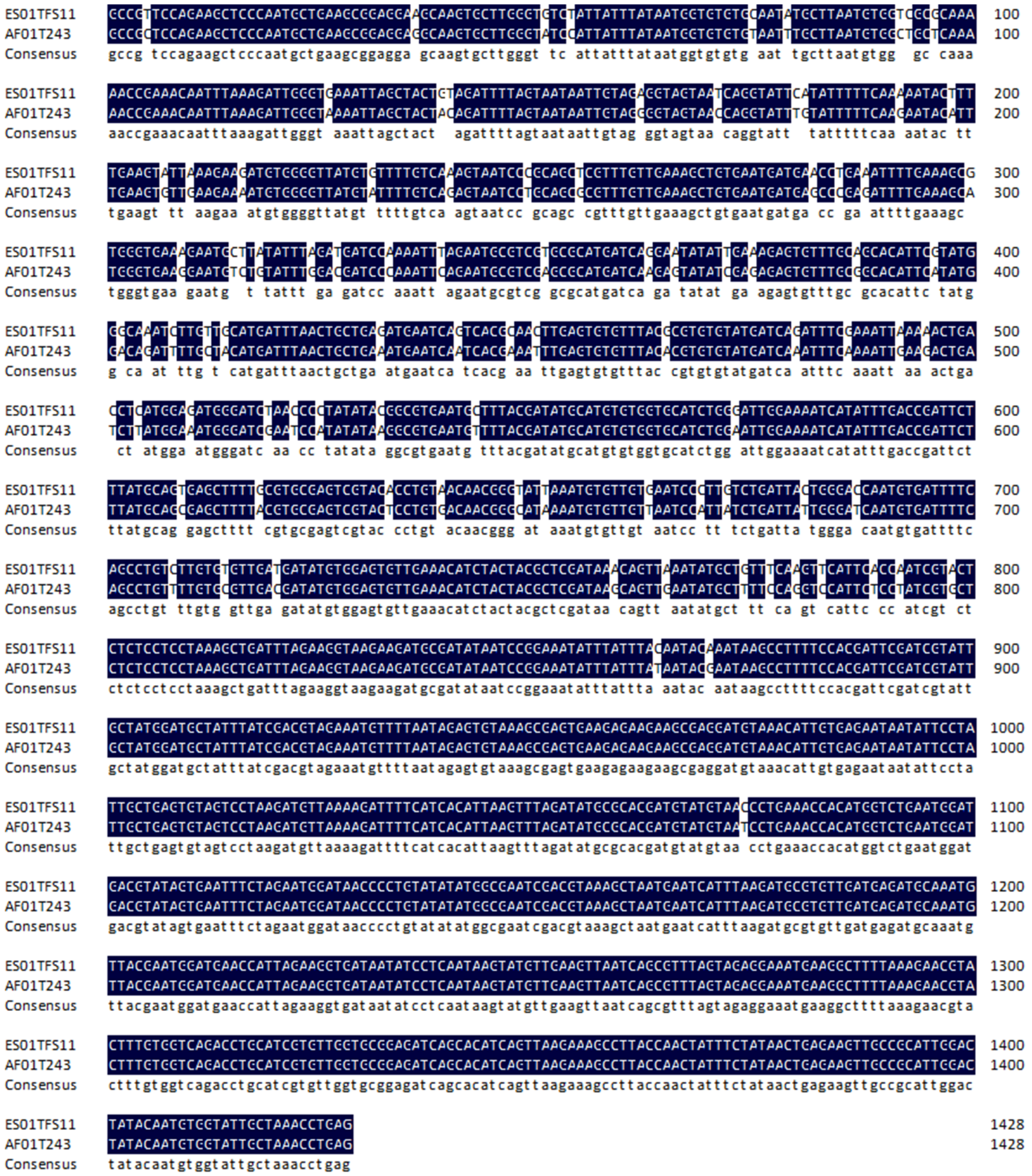

Figure S4: Pairwise alignment of DWV helicase encoding domain between ES01TFS11 and AF01T243

Table S1. Detail information of collection

Species/Sample soure	Date (Y/M/D)	District *	Longitude and latitude	
			X (TWD97)	Y (TWD97)
Apis mellifera, Apis florea<br>Open space				
	2020/09/25	Qianzhen[a]	180315.42	2499557.03
	2020/09/25	Qianzhen	179254.60	2498733.59
	2020/09/25	Qianzhen	179670.04	2499409.38
	2020/09/25	Lingya	177800.73	2501163.26
	2020/11/27	Gangshan	184338.79	2501697.48
	2020/11/27	Fengshan	184995.21	2501517.24
	2020/11/27	Fengshan	182106.54	2502329.55
	2021/03/05	Qijin	175341.05	2500305.85
	2021/03/19	Gushan	176788.31	2506311.87
	2021/04/01	Qijin[b]	175383.35	2500299.39
	2021/04/01	Qianzhen[a]	180315.42	2499557.03
	2021/04/16	Fengshan	182106.55	2502329.55
	2021/05/14	Gushan	174501.87	2502629.29
	2021/05/14	Qijin[b]	175383.35	2500299.39
	2021/12/15	Qijin[b]	175383.35	2500299.39
Apis florea, Euvarroa sinhai<br>Nest				
	2020/05/05	Xiaogang	181347.45	2496728.64
	2020/05/15	Qianzhen	178289.98	2501132.17
	2020/06/06	Qianzhen	178601.23	2500853.88
	2020/07/11	Qianzhen	180426.96	2501130.13
	2020/07/20	Qianzhen	180500.95	2500108.75
	2021/04/10	Fengshan	182083.04	2499924.82
	2021/05/14	Gushan	176678.83	2506443.73
	2021/05/30	Lingya	180364.69	2502904.70
	2021/07/01	Lingya	180743.79	2504155.12
	2021/08/25	Xiaogang	183106.76	2496646.63
	2021/12/15	Qijin	176568.03	2499001.46
	2022/01/01	Gushan	176678.83	2506443.73
	2022/01/31	Lingya	179354.77	2502919.72
Varroa destructor<br>Nest				
	2021/06/14	NCYU (CHAYI)	197368.08	2596168.14

*,**: the same alphabetical symbols represented sample collected from same localityTable S2.

Sequence of the two novel isolates, AF01T243 and ES01TFS11

Isolate: AF01T243

accession number: OP889266

atggcttttagttgtggcgctctttcttattctgctgttgctcaagccccttctgtcccttattcacctcgtgcatgggaagtcgatgaagctaggagacgt
cgtgtaattaaacgtttggcgttggaacaggaacgtgttcgaaacgttcttgatgttgatgtttataatcaagcgacatgggaacaggaagatgagc
gtgacaatgaatttttgacggaacagttaaacaatttatatactatttactcgatagctgaacgttgtgtgcgtcggcctattgtggaacatgtccctata
tcggttatgaatcggttttctcctttggaatctcttaacattgaaattggtcaagaagcaggcgaatgtatatttaagaagcctaagtatacgcgcgttt
gtaagaaagttaagcgagttacttctcgatttgttcgcgaaagggttatccgccccgtgtgtagtaggtcacccatgttattatttaaacttaagaaagc
tatttatgatttacatttatatagattaagaaaacagattaggatattaaggtaccaaaaacaacgtgattatgaattagagtgtgcatctagtctgttac
aattagctaatccggtgcaggctaatccggagatggataatcctaatcctggacctgatggtgaaggtgaagtagagctagaaaaagatagtaatg
ttgtgttgacaactcaacgtgatcctagtacttctattccagcacctatcagtgttaaatggagtaggtggactagtaatgacgtggttgatgattatgc
taccattacatctcggtggtaccagattgcagaatttgtttggtcaaaggatgatccgtttgataaagaattagcgcgtttaattttgcctcgtgctttatt
atctagcattgaagagaactcagatgccatatgcgatgtacctaacacaataccttttaaagtgcatgcttattggcgaggtgatatggaagtaagag
ttcaaatcaattcaaataaatttcaagtcggtcaattacaagcgacatggtattattcggaccatgaaaatctaaacatgtcgtcaaaaagaagtgtgt
atggattttcacagatggatcatgctttgattagtgcatccgcaagtaatgaagcgaaattagttattccgtttaaacatgtttatccatttttaccaacaa
gagtcgtaccagattggactacgggtattttagatatgggtactttaaacattcgtgtgatagctcctttacggatgagtgctactggtcctactacttgt
aatgttgttgtatttattaaattaaataatagcgaatttacaggtacttcctctggcaagttttatgaaaatcaagttaaggcaaaacctgagatggatc
gtatactaaatctggcagaaggattattgaacaacacaattggaggtaataatatggataatccttcttatcaacaatctccccgtcattttgttccgacc
ggtatgcatagtttagcattaggtactaatttagtagaaccattacacgcactacgtctagatgcggctggtactacacaacatccggtaggttgtgcg
cctgatgaagatatgactgtatcttctattgcatctcgttatggcttgattaggcaagttcaatggaagaaagatcatgcaaaagggtcgcttttattgc
aacttgatgcggatccatttgtagaacagaaaattgaaggttcaaacccaatatctttgtattggtttgcacctgttggtgttgtgtctagtatgttcatg
caatggagaggatcattagaatataggtttgatattatagcatctcaatttcataccggtaggcttatagtaggttatgttcctggtttaacaacgacttt
gcaagagaaaatggattatatgaaattaaaatcatcaagttatgtggtatttgatttacaggaaagtaatagttttacttttgaggtaccatatgtttcat
atagaccatggtgggtgcgtaaatatggtggtaattatttgccttcatcgactgatgctcctagtacattgtttatgtatgtgcaagttccattaatacct
tggaagctgtttcggacaccatagatattaatgtatatgtgcgtgggggtagttcttttgaagtttgtgtaccggttcagcctagtttaggcttaaattg
gaatacagactttatattgcgtaatgatgaagaatatagggctaagactggttatgcgccatactatgcgggtgtgtggcatagctttaataatagta
attctcttgtttttagatggggatctgcttctgatcaaattgctcaatggccaactattactgtacctaaaggtgaacttgcatttttacggattaaagatg
cgaaacaagccgctgttggaaatcaaccgtggcgtgcaatggttgtttggccttctggtcatggttataatattggtataccaacatataacgctgagc
gagcacgtcagcttgctcaacatttatatgggggtggatcattaactgatgatagggcgaaacaactatttgttcctgctagtcaacaaggacctggt
acttatagtaaaggcaatccagtatgggaagtcatgcgagcaccgttggcaactcagcgtgcgcaagttcaagattttgaatttattgaagctatgcc
tgaaggagaagaatctcgtaatactacggttttggacacaaccactgctttacaatctagtggatttggtcgtgcctttttcggagaagcatttaatgat
ctcaaaacgttaatgcggagatatcaattgtatggtcagttattattgtctgttactacggacaaagatatcgatcattgtatgtttaccttcccttgtttac
cgcaagggttgtcgttagacattggttccgccggagcaccacatgaaatctttaatagatgtcgtgatggtattattcctttaattgcatcggggtatag
attttatagaggagatctccgttataagattgtatttcctagtaatgttaatagtaatatatgggttcaacatcgacccgaccgtaggttagagggatgg
tctgcagcgaaaattgtaaattgtgatgctgtgtctactggtcaaggagtatataatcatggttacgctagccatattcagatcacgcgagtaaataat
gttatagaattagaagttccattttataatgcgacttgctacaattatttgcaagcatttcaagcatccagcgctgcatctagttatgcagtatctttagga
gaaatatcggttggttttcaagctacaagtgatgatattgcatctattgttaacaaacctgttactatttattatagtataggagatggtatgcaattttctc
agtgggttggatatcaaccgatgatgatcctagaccagcttcctgcaccagtagtaagggccgtgcctgagggccctattgcgaagattaaaaactt

cttccatcaaacagctgacgaggttagagaagctcaggcagcaaagatgcgtgaagatatgggtatggttgtccaagatgttattggagaacttag
ccaggccataccggatcttcaacaaccagaggttcaagcaaatgtcttctcactggtgtctcagttagtgcatgctattataggtactagtttgaagac
agttgcttgggcaattgtttcgatttttgtgactttaggattaattggacgcgaaatgatgcattcagttattactgtagtcaagcggttacttgagaaat
atcacttggcgacgcaaccccaggaatccgccaattcaagtactgttatatctgccgctccagaagctcccaatgctgaagcggaggaggcaagtg
cttgggtatccattatttataatggtgtgtgtaatttgcttaatgtggctgctcaaaaaccgaaacaatttaaagattgggtaaaattagctactacagat
tttagtaataattgtaggggtagtaaccaggtatttgtatttttcaagaatacatttgaagtgttgaagaaaatgtggggttatgtattttgtcagagtaa
tcctgcagcgcgtttgttgaaagctgtgaatgatgagcccgagattttgaaagcatgggtgaaggaatgtctgtatttggacgatcccaaattcaga
atgcgtcgagcgcatgatcaagagtatatcgagagagtgtttgcggcacattcatatggacagattttgctacatgatttaactgctgaaatgaatca
atcacgaaatttgagtgtgtttacacgtgtgtatgatcaaatttcaaaattgaagactgatcttatggaaatgggatcgaatccatatataaggcgtga
atgttttacgatatgcatgtgtggtgcatctggaattggaaaatcatatttgaccgattctttatgcagcgagcttttacgtgcgagtcgtactcctgtga
caacgggcataaaatgtgttgttaatccattatctgattattgggatcaatgtgattttcagcctgttttgtgcgttgacgatatgtggagtgttgaaaca
tctactacgctcgataagcagttgaatatgcttttccaggtccattctcctatcgtgctctctcctcctaaagctgatttagaaggtaagaagatgcgata
taatccggaaatatttatttataatacgaataagccttttccacgattcgatcgtattgctatggatgctatttatcgacgtagaaatgttttaatagagtg
taaagcgagtgaagagaagaagcgaggatgtaaacattgtgagaataatattcctattgctgagtgtagtcctaagatgttaaaagattttcatcac
attaagtttagatatgcgcacgatgtatgtaatcctgaaaccacatggtctgaatggatgacgtatagtgaatttctagaatggataacccctgtatat
atggcgaatcgacgtaaagctaatgaatcatttaagatgcgtgttgatgagatgcaaatgttacgaatggatgaaccattagaaggtgataatatcc
tcaataagtatgttgaagttaatcagcgtttagtagaggaaatgaaggcttttaaagaacgtactttgtggtcagacctgcatcgtgttggtgcggag
atcagcacatcagttaagaaagccttaccaactatttctataactgagaagttgccgcattggactatacaatgtggtattgctaaacctgagatggat
caagcttatgaggttatgagttcatatgcagccggaatgaatgcagaaatcgaagcacatgagcaaattcggcgtttgtcagtagaatgccaatac
gcagaaccacaagccacaagaaattttgaggataatgaaccaactatagatgaggaacttatgggcgacgctgaatttacatcacaagctttagaa
cgtcttgtggatgaaggttatataactggaaaccagaagaaatatatagctgcatggtgtagtaagcgtcgagaacacgttgctgattttgatctcatt
tggactgataatttgcgagtattaagtgcatacgcgcatgaacgctcatctgcgacacgactttctacggatgatgttaaattgtataagacgattagt
atgttacatcaaaaatatgataccacggagtgtgctaaatgccaacattggtatgctcctttaacagcaatttacgtagatgataagaaattattttggt
gccaaaaagaaaagaagacgctcattgatactcgaaagttgtcgaaagaagacgtgactgttcaatcaaaattgattaacttgtctattccttgtgga
gaagtgtgtatgttacattctaaatattttaattatcttttccataaagcgtggttgtttgagaacccaacttggcgcctgatatacaatggcaccaagaa
gggtatgcctgaatatttcatgaactgtgtggatgaaatttcattagattcaaaatttggacaggtgaaagtatggttacaaaccataattgataaatat
ttaactcgtcctattaaaatgatacgtgatttcttgtttaaatggtggccgcaagtggcatatgtattaagtttgttaggtataattggtattacagcctat
gaaactaagagtcctaagccgtcttcagaagagttagtagaccactatgtgaataaacattgtagttctgacttttggtcatcaggaatggcaacacct
caagggttgaagtatagtgaagcggtaacagctaaaacgcctaggattcataggttgccggttactactaggcctcaaggatcaacacaacaagta
gacgctgcagtaaataaaatattgcagaatatggttaatattggcgttgtttttccaaaggtacctggtagtaagtggcgagatattaattttagatgct
taatgcttcataataggcaatgtttaatgttaaggcattatattgagtcgactgcttctttccctgctggaactaagtattatttcaagtatattcataatca
agaaactagaatgtcaggtgacatttctgggattgaaattgatttattaaatttacctagattatactatggtggtcttgcaggagaggaatcgtttgac
agcaatatagtgcttgtgactatgccgaaccgtattcctgagtgtaagagtattgttaaatttatagcttcacatagcgagcatatgcgtgctcagaat
gatggtgtattagtaactggagaacatactcagttgttggcattcgagaacaataataagacgccaataagcattaacgctgatggtttgtatgaggt
tatacttcaaggagtatatacttatccttaccatggtgatggtgtttgtggatctatattgttgtctcgaaatttacagcgaccgattattggcattcatgtt
gctggtacagagggattgcatggttttggcgttgctgaaccacttgtacatgagatgtttactggcaaagcaattgagagtgagagagaaccgtatg
accgtgtgtatgaattacctctgcgtgaattagatgagtctgatattggtttagatactgatttatatccgattggtagagttgatgcaaagttagctcat
gctcaaagtccttctactgggattaaaaagacgcttattcatggaacatttgatgttaggacagaaccaaatcctatgtcatcgcgtgatccgagaata

gcgccatatgatcctctaaaattgggatgtgaaaagcatggtatgccttgctcaccgtttaataggaaacatctggaattagcgactactcatttgaaa
gaaaaattaatctcagtagttaaaccaataaatggttgtaagataagaagtttgcaggatgctgtgtgtggtgtgcctggtttggatggttttgattcta
tatcttggaatactagtgcaggtttccctttgtcttcattgaaaccacctggaacatcaggaaaacgatggttgtttgatattgaacttcaggatacagg
atgttatttattgcgtggaatgcgtcctgaacttgagattcaattatcaacgactcagttaatgaggaagaagggaataaagcctcacactatattcac
tgactgcttgaaagatacttgtttacctgttgaaaaatgtaggatacctggtaagactagaatatttagtataagtcctgtacagtttactattccatttcg
acaatactatcttgattttatggcatcttatcgagcagcgcgacttaatgctgaacatgggattggcatagatgttaacagcttagaatggactaattta
gcgacaagtttgtctaaatacggtactcatattgtgacgggtgactataagaactttggtcctggattagattccgatgttgctgcttctgcgtttgagat
tatcattgattgggtgttacattataccgaagaagatgataaggatgagatgaagcgtgtgatgtggacaatggcgcaagaaattcttacgcctagt
catctatgtcgtgatttggtgtaccgggtaccttgtggaattccatcaggttctccaataaccgacatattgaataccatttcaaattgtctgttaataagg
ttagcttggttaggtattactgatcttcccttgtctgaattctctcaaaatgttgttcttgtttgttatggtgatgatcttatcatgaatgttagtgataacatg
attgaaaagtttaatgctgtaacaataggaaatttcttttcacaatataagatggaatttacggatcaagataaatcagggaatactgtaaagtggcg
aacattacaaactgccactttcttgaagcacgggtttttaaaacatccaactagacctgtgtttttagctaacctcgataaggtatcagtggaaggtacg
acgaattggactcatgctcgaggattgggtcgtcgcacagcaacgatagagaatgctaagcaagcgctagaattagcgtttggttggggtcctgaa
tacttcaactatgtcagaaacaccattaaaatggcatttgataaattgggtatttacgaagatctcatcacttgggaagaaatggatgttagatgttatg
ccaatgcg

Isolate: ES01TFS11

accession number: OP889267

---

atggcttttagttgtggcgctctttcttattctgctgttgctcaagccccttctgtcccttattcacctcgtgcatgggaagtcgatgaagctaggagacgt
cgtgtaattaaacgtttggcgttggaacaggaacgtgttcgaaacgttcttgatgttgatgtttataatcaagcgacatgggaacaggaagatgagc
gtgacaatgaatttttgacggaacagttaaacaatttatatactatttactcgatagctgaacgttgtgtgcgtcggcctattgtggaacatgtccctata
tcggttatgaatcggttttctcctttggaatctcttaacattgaaattggtcaagaagcaggcgaatgtatatttaagaagcctaagtatacgcgtgtttg
taagaaagttaagcgagttacttctcgatttgttcgcgaaagggttatccgccccgtgtgtagtaggtcacccatgttattatttaaacttaagaaagct
atttatgatttacatttatatagattaagaaaacagattaggatattaaggtaccaaaaacaacgtgattatgaattagagtgtgcatctagtctgttac
aattagctaatccagtgcaagctaagccggagatggataatcctaatcctggacctgatggtgaaggtgaagtagagctagaaaaagatagtaat
gttgtgttgacaactcaacgtgatcctagtacttctattccagcacctatcagtgttaaatggagtaggtggactagtaatgacgtggttgatgattatg
ctaccattacatctcggtggtaccagattgcagaatttgtttggtcaaaggatgatccgtttgataaagaattagcgcgtttaattttgcctcgtgctttat
tatctagcattgaagagaactcagatgccatatgcgatgtacctaacacaataccttttaaagtgcatgcttattggcgaggtgatatggaagtaaga
gttcaaatcaattcaaataaatttcaagtcggtcaattacaagcgacatggtattattcggaccatgaaaatctaaacatgtcgtcaaaaagaagtgtg
tatggattttcacagatggatcatgctttgattagtgcatccgcaagtaatgaagcgaaattagttattccgtttaaacatgtttatccatttttaccaaca
agagtcgtaccagattggactacgggtattttagatatgggtactttaaacattcgtgtgatagctcctttacggatgagtgctactggtcctactactt
gtaatgttgttgtatttattaaattaaataatagcgaatttacaggtacttcctctggcaagttttatgaaaatcaagttaaggcaaaacctgagatggat
cgtatactaaatctggcagaaggattattgaacaacacaattggaggtaataatatggataatccttcttatcaacaatctccccgtcatttcgttccga
ccggtatgcatagtttagcattaggtactaatttagtagaaccattacacgcactacgtctagatgcggctggtactacacaacatccggtaggttgtg
cgcctgatgaagatatgactgtatcttctattgcatctcgttatggcttgattaggcaagttcaatggaagaaagatcatgcaaaagggtcgcttttatt
gcaacttgatgcggatccatttgtagaacagaaaattgaaggttcaaacccaatatctttgtattggtttgcacctgttggtgttgtgtctagtatgttca
tgcaatggagaggatcattagaatataggtttgatattatagcatctcaatttcataccggtaggcttatagtaggttatgttcctggtttaacaacgact
ttgcaagagaaaatggattatatgaaattaaaatcatcaagttatgtggtatttgatttacaggaaagtaatagttttacttttgaggtaccatatgtttc
atatagaccatggtgggtgcgtaaatatggtggtaattatttgccttcatcgactgatgctcctagtacattgtttatgtatgtgcaagttccattaatacc

---

tatggaagctgtttcggacaccatagatattaatgtatatgtgcgtgggggtagttcttttgaagtttgtgtaccggttcagcctagtttaggcttaaatt
ggaatacagactttatattgcgtaatgatgaagaatatagggctaagactggttatgcgccatactatgcgggtgtgtggcatagctttaataatagt
aattttcttgtttttagatggggatctgcttctgatcaaattgctcaatggccaactattactgtacctaaaggtgaacttgcatttttacggattaaagat
gcgaaacaagccgctgttggaaatcaaccgtggcgtgcaatggttgtttggccttctggtcatggttataatattggtataccaacatataacgctga
gcgagcacgtcagcttgctcaacatttatatgggggtggatcattaactgatgatagggcgaaacaactatttgttcctgctagtcaacaaggacctg
gtacttatagtaaaggcaatccagtatgggaagtcatgcgagcaccgttggcaactcagcgtgcgcaagttcaagattttgaatttattgaagctatg
cctgaaggagaagaatctcgtaatactacggttttggacacaaccactgctttacaatctagtggatttggtcgtgcctttttcggagaagcatttaatg
atctcaaaacgttaatgcggagatatcaattgtatggtcagttattattgtctgttactacggacaaagatatcgatcattgtatgtttaccttcccttgttt
accgcaagggttgtcgttagacattggttccgccggagcaccacatgaaatctttaatagatgtcgtgatggtattattcctttaattgcatcggggtat
agattttatagaggagatctccgttataagattgtatttcctagtaatgttaatagtaatatatgggttcaacatcgacccgaccgtagattagagggat
ggtctgcagcgaaaattgtaaattgtgatgctgtgtctactggtcaaggagtatataatcatggttacgctagccatattcagatcacgcgagtaaata
atgttatagaattagaagttccattttataatgcgacttgctataattatttgcaagcatttcaagcatccagtgctgcgtcaagttatgcagtatcgttag
gagaaatatcggttggttttcaggccacgagcgatgacattgcaactattgtaaataagcctgtaactatttattatagtataggtgatggcatgcaatt
ttcgcagtgggttggatatcaaccgatgatgattttagaccaacttccagcgccagtagttaaggcagtgcctgagggccctattgcaaaaattaaaa
atttctttcatcaaacagctgatgaggtgagagaagctcaggcagcaaagatgcgggaagatatgggtgtagttgttcaggatgttattggagaac
ttagtcaggctataccggatcttcagcaaccagaagttcaagctaatgttttctcattagtgtctcaattagtgcatgctattataggtactagtttgaaa
acggtggcttgggcaattgtttcaatttttgtgactttaggattgattggacgcgaaatgatgcattcagtcattactgtggtcaaacgactacttgaga
aatatcacttggcgacgcaaccccaggaatccgccaattcaagtactgttatatcagccgttccagaagctcccaatgctgaagcggaggaagcaa
gtgcttgggtgtctattatttataatggtgtgtgcaatatgcttaatgtggtcgcgcaaaaaccgaaacaatttaaagattgggtgaaattagctactgt
agattttagtaataattgtagaggtagtaatcaggtattcatatttttcaaaaatacttttgaagtattaaagaagatgtggggttatgtgttttgtcaaa
gtaatcccgcagctcgtttgttgaaagctgtgaatgatgaacctgaaattttgaaagcgtgggtgaaagaatgcttatatttagatgatccaaaattta
gaatgcgtcgtgcgcatgatcaggaatatattgaaagagtgtttgcagcacattcgtatgggcaaatcttgttgcatgatttaactgctgagatgaatc
agtcacgcaacttgagtgtgtttacgcgtgtgtatgatcagatttcgaaattaaaaactgacctcatggagatgggatctaacccctatatacggcgtg
aatgctttacgatatgcatgtgtggtgcatctgggattggaaaatcatatttgaccgattctttatgcagtgagcttttgcgtgcgagtcgtacacctgta
acaacgggtattaaatgtgttgtgaatcccttgtctgattactgggaccaatgtgattttcagcctgtcttgtgtgttgatgatatgtggagtgttgaaac
atctactacgctcgataaacagttaaatatgctgtttcaagttcattcaccaatcgtactctctcctcctaaagctgatttagaaggtaagaagatgcgat
ataatccggaaatatttatttacaatacaaataagccttttccacgattcgatcgtattgctatggatgctatttatcgacgtagaaatgttttaatagagt
gtaaagcgagtgaagagaagaagcgaggatgtaaacattgtgagaataatattcctattgctgagtgtagtcctaagatgttaaaagattttcatca
cattaagtttagatatgcgcacgatgtatgtaaccctgaaaccacatggtctgaatggatgacgtatagtgaatttctagaatggataacccctgtata
tatggcgaatcgacgtaaagctaatgaatcatttaagatgcgtgttgatgagatgcaaatgttacgaatggatgaaccattagaaggtgataatatc
ctcaataagtatgttgaagttaatcagcgtttagtagaggaaatgaaggcttttaaagaacgtactttgtggtcagacctgcatcgtgttggtgcgga
gatcagcacatcagttaagaaagccttaccaactatttctataactgagaagttgccgcattggactatacaatgtggtattgctaaacctgagatgga
tcaagcttatgaggttatgagttcatatgcagccggaatgaatgcagaaatcgaagcacatgagcaaattcggcgtttgtcagtagaatgccaatac
gcagaaccacaagccacaagaaattttgaggataatgaaccaactatagatgaggaacttatgggcgacgctgaatttacatcacaagctttagaa
cgtcttgtggatgaaggttatataactggaaaccagaagaaatatatagctgcatggtgtagtaagcgtcgagaacacgttgctgattttgatctcatt
tggactgataatttgcgagtattaagtgcatacgcgcatgaacgctcatctgcgacacgactttctacggatgatgttaaattgtataagacgattagt
atgttacatcaaaaatatgataccacggagtgtgctaaatgccaacattggtatgctcctttaacagcaatttacgtagatgataagaaattattttggt
gccaaaaagaaaagaagacgctcattgatactcgaaagttgtcgaaagaagacgtgactgttcaatcaaaattgattaacttgtctattccttgtgga

gaagtgtgtatgttacattctaaatattttaattatcttttccataaagcgtggttgtttgagaacccaacttggcgcctgatatacaatggcaccaagaa
gggtatgcctgaatatttcatgaactgtgtggatgaaatttcattagattcaaaatttggacaggtgaaagtatggttacaaaccataattgataaatat
ttaactcgtcctattaaaatgatacgtgatttcttgtttaaatggtggccgcaagtggcatatgtattaagtttgttaggtataattggtattacagcctat
gaaactaagagtcctaagccgtcttcagaagagttagtagaccactatgtgaataaacattgtagttctgacttttggtcatcaggaatggcaacacct
caagggttgaagtatagtgaagcggtaacagctaaaacgcctaggattcataggttgccggttactactaggcctcaaggatcaacacaacaagta
gacgctgcagtaaataaaatattgcagaatatggttaatattggcgttgtttttccaaaggtacctggtagtaagtggcgagatattaattttagatgct
taatgcttcataataggcaatgtttaatgttaaggcattatattgagtcgactgcttctttccctgctggaactaagtattatttcaagtatattcataatca
agaaactagaatgtcaggtgacatttctgggattgaaattgatttattaaatttacctagattatactatggtggtcttgcaggagaggaatcgtttgac
agcaatatagtgcttgtgactatgccgaaccgtattcctgagtgtaagagtattgttaaatttatagcttcacatagcgagcatatgcgtgctcagaat
gatggtgtattagtaactggagaacatactcagttgttggcattcgagaacaataataagacgccaataagcattaacgctgatggtttgtatgaggt
tatacttcaaggagtatatacttatccttaccatggtgatggtgtttgtggatctatattgttgtctcgaaatttacagcgaccgattattggcattcatgtt
gctggtacagagggattgcatggttttggcgttgctgaaccacttgtacatgagatgtttactggcaaagcaattgagagtgagagagaaccgtatg
accgtgtgtatgaattacctctgcgtgaattagatgagtctgatattggtttagatactgatttatatccgattggtagagttgatgcaaagttagctcat
gctcaaagtccttctactgggattaaaaagacgcttattcatggaacatttgatgttaggacagaaccaaatcctatgtcatcgcgtgatccgagaata
gcgccatatgatcctctaaaattgggatgtgaaaagcatggtatgccttgctcaccgtttaataggaaacatctggaattagcgactactcatttgaaa
gaaaaattaatctcagtagttaaaccaataaatggttgtaagataagaagtttgcaggatgctgtgtgtggtgtgcctggtttggatggttttgattcta
tatcttggaatactagtgcaggtttccctttgtcttcattgaaaccacctggaacatcaggaaaacgatggttgtttgatattgaacttcaggatacagg
atgttatttattgcgtggaatgcgtcctgaacttgagattcaattatcaacgactcagttaatgaggaagaagggaataaagcctcacactatattcac
tgactgcttgaaagatacttgtttacctgttgaaaaatgtaggatacctggtaagactagaatatttagtataagtcctgtacagtttactattccatttcg
acaatactatcttgattttatggcatcttatcgagcagcgcgacttaatgctgaacatgggattggcatagatgttaacagcttagaatggactaattta
gcgacaagtttgtctaaatacggtactcatattgtgacgggtgactataagaactttggtcctggattagattccgatgttgctgcttctgcgtttgagat
tatcattgattgggtgttacattataccgaagaagatgataaggatgagatgaagcgtgtgatgtggacaatggcgcaagaaattcttacgcctagt
catctatgtcgtgatttggtgtaccgggtaccttgtggaattccatcaggttctccaataaccgacatattgaataccatttcaaattgtctgttaataagg
ttagcttggttaggtattactgatcttcccttgtctgaattctctcaaaatgttgttcttgtttgttatggtgatgatcttatcatgaatgttagtgataacatg
attgaaaagtttaatgctgtaacaataggaaatttcttttcacaatataagatggaatttacggatcaagataaatcagggaatactgtaaagtggcg
aacattacaaactgccactttcttgaagcacgggtttttaaaacatccaactagacctgtgtttttagctaacctcgataaggtatcagtggaaggtacg
acgaattggactcatgctcgaggattgggtcgtcgcacagcaacgatagagaatgctaagcaagcgctagaattagcgtttggttggggtcctgaa
tacttcaactatgtcagaaacaccattaaaatggcatttgataaattgggtatttacgaagatctcatcacttgggaagaaatggatgttagatgttatg
ccaatgcg
